# Shaping chromatin with DICER

**DOI:** 10.18632/oncotarget.17773

**Published:** 2017-05-10

**Authors:** Shalaka Chitale, Holger Richly

**Affiliations:** Laboratory of Molecular Epigenetics, Institute of Molecular Biology, Ackermannweg, Mainz, Germany

**Keywords:** epigenetics, chromatin dynamics, DICER

**Figure 1:**
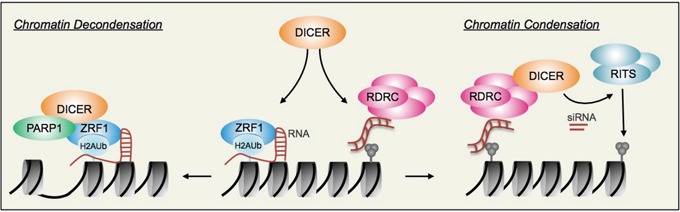
Regulating chromatin conformation: The two faces of DICER DICER facilitates *chromatin decondensation* by assembling with ZRF1 and PARP1. ZRF1 tethers to chromatin that is mono-ubiquitylated at lysine 119 of histone H2A and RNA species contribute to ZRF1 occupancy at chromatin. ZRF1 provides a binding platform and recruits DICER to chromatin. Multiprotein complexes consisting of DICER, ZRF1, PARP1 and potentially proteins from the family of SWI/SNF chromatin remodelling complexes cause a local decondensation of chromatin. Further, DICER assembles with RNA-dependent RNA polymerase complexes (RDRC) and facilitates *chromatin condensation* by targeting RITS complexes to chromatin thereby spreading H3K9 methylation (H3K9me3; grey balls) to silence chromatin. For details refer to [3].

The accessibility of chromatin is an essential feature of DNA templated processes such as DNA replication, transcription and DNA repair. The chromatin condensation state, and thus accessibility, is modulated by a variety of chromatin remodeling complexes [1] as well as epigenetic components; for instance by bridging gene regulatory elements [2]. More recently, the function of ncRNAs in regulating chromatin has received much attention. One important player that processes ncRNAs to control gene expression in the cytoplasm and nucleus is the endoribonuclease DICER. DICER cleaves double-stranded RNA into short double-stranded RNA fragments called small interfering RNAs (siRNA) and microRNA (miRNA). Whereas miRNAs are essential to degrade mRNAs in the cytoplasm, siRNAs produced by DICER contribute to the generation of heterochromatin [3] (Figure 1). RNA generated from the locus to be silenced is converted into dsRNA by the activity of RNA dependent RNA polymerase (RDRP), and the resulting dsRNA is subsequently processed by DICER (Figure 1). siRNAs generated by DICER are loaded into the RITS (RNA-induced transcriptional silencing) complex, which targets the RITS complex to chromatin causing the spreading of H3K9-trimethylation along the chromatin fiber [3]. Likewise, DICER contributes to the DNA damage response by generating small non-coding RNAs at DNA double strand breaks that are necessary for the recruitment of the mediators MDC1 and 53BP1 [4]. 

Very recently a novel function of DICER was identified that contrasts its conventional RNA-dependent functions in regulating chromatin conformation [5]. Contrary to its role in forming heterochromatin in a siRNA dependent fashion, DICER also facilitates the decondensation of chromatin at DNA damage sites. Importantly, this novel and unexpected role was demonstrated to be independent of its catalytic activity. DICER is recruited to chromatin by the H2A-ubiquitin binding protein ZRF1, which links DICER not only to the ubiquitin signaling pathways during DNA repair but potentially also to the ZRF1-dependent activation of genes during stem cell differentiation. ZRF1 associates to chromatin by tethering to the epigenetic H2A-ubiquitin mark and by binding RNA with its c-terminal SANT domains [6] (Figure 1). ZRF1 may be regarded a tethering platform for DICER, the Poly [ADP-ribose] polymerase 1 (PARP1) and SWI/SNF chromatin remodelling complexes [5], which contribute to the decondensation of chromatin structures. Hence, DICER represents a general tool to shape the chromatin conformation. Employing its siRNA processing activity it contributes to chromatin condensation, while in complex with ZRF1 and chromatin remodeling factors and independent of its catalytic activity it contributes to chromatin decondensation (Figure 1). An important question remaining is how DICER carries out these different functions and in particular how it can facilitate chromatin decondensation. Regulating the association of DICER with chromatin may be a potential mechanism of modulating its activity. DICER harbors two helicase domains, which catalyze the separation of double stranded nucleic acids. This function, apart from acting on dsRNAs, might also contribute to altering the confomation of DNA. However, the primary mode of action of DICER appears to involve the assembly of chromatin remodelling complexes. PARP1 inhibitors as well as a knockdown of PARP1 entirely abolish DICER-dependent chromatin decondensation further supporting this premise [5]. DICER contains a PAZ domain at its c-terminus, which interacts with PIWI, Argonaut and Zwille. Although a similar interaction of the PAZ domain with PARP1 or SWI/SNF complexes is yet to be identified, mutually exclusive association of the PAZ domain with either chromatin remodellers or the aforementioned proteins might be a potential mechanism of modulating DICER activity. Interaction with the RNA-dependent pathway (PIWI, Argonaut, Zwille) would lead to the generation of heterochromatin, while interaction with an RNA-independent mechanism (PARP1, SWI/SNF) would cause decondensation. Future work needs to address the underlying mechanisms to shed light on DICER pathway choice. In a larger scope, DICER mediated chromatin decondensation might be required during other chromatin-associated processes. The recruitment of DICER relies on ZRF1 and hence H2A-ubiquitylation at lysine 119 providing a tethering platform for ZRF1. This epigenetic mark is essential for transcriptional activation of Polycomb-repressed genes during differentiation of embryonic stem cells [7]. Depletion of ZRF1 causes abnormal differentiation and impairs formation of all three germ layers during embryonic development. Given the robust interaction of DICER with ZRF1 [5], DICER, apart from its cytoplasmic function in conjunction with the RISC complex, might turn out to be a master regulator of chromatin conformation during cellular differentiation. Likewise, DNA replication is accompanied by H2A-ubiquitylation, which could constitute another example for ZRF1-DICER facilitated chromatin decondensation.

Without doubt, DICER has entered the scene as a regulator of chromatin organization. It is now time to unveil its versatile functions to better understand the dynamic changes of the chromatin conformation.
